# The impact of magnitude and duration of plasma viremia during analytical treatment interruptions on CD4^+^ T cell recovery after ART resumption

**DOI:** 10.1016/j.jve.2025.100604

**Published:** 2025-06-27

**Authors:** Vibeke Klastrup, Jesper Damsgaard Gunst, Ole Schmeltz Søgaard

**Affiliations:** a Department of Clinical Medicine, Aarhus University, Denmark; b Department of Infectious Diseases, Aarhus University Hospital, Denmark

**Keywords:** HIV-1, Cure, ATI, Plasma viral load, CD4^+^ T cell recovery

## Abstract

**Objective:**

Analytical treatment interruption (ATI) is crucial for assessing the efficacy of HIV-1 cure strategies. Recent cure studies have implemented more flexible ART restart criteria, permitting higher plasma viral loads (pVLs) for longer periods, which could potentially impair CD4^+^ T cell recovery even following ART resumption.

**Design:**

We conducted a pooled analysis of six clinical HIV cure trials that included an ATI to evaluate the impact of magnitude and duration of plasma viremia during ATI on CD4^+^ T cell dynamics.

**Methods:**

Wilcoxon signed-rank or rank-sum test was used to analyze differences in CD4^+^ T cell counts from 3 time points: 1) pre-ATI, 2) ART resumption, and 3) ART-induced viral re-suppression, with analyses stratified by peak pVL (≤10,000 or >10,000 copies/mL) or by duration of viremia (0–14, 15–28, or >28 days).

**Results:**

Among 114 participants, we found no change in CD4^+^ T cell counts from pre-ATI to post-ATI (at viral re-suppression, *P* = 0.80). We also found no impact of low (≤10,000 copies/mL) versus high (>10,000 copies/mL) peak viremia on CD4^+^ T cell counts at the time of ART resumption or viral re-suppression (*P* = 0.48, *P* = 0.88, respectively). Similarly, the change in CD4^+^ T cell count from pre-ATI to viral re-suppression did not differ significantly between individuals with viremia lasting 0–14 days versus those with >28 days.

**Conclusion:**

In our pooled analysis, high peak rebound pVLs and longer duration of viremia did not impair CD4^+^ T cell recovery following the resumption of ART, supporting the safety of more flexible ATIs in HIV-1 cure trials.

## Teaser

Summary: The levels and duration of plasma HIV-1 RNA during ATI do not impact CD4^+^ T cell recovery after ART resumption.

## 1 Introduction

Antiretroviral therapy (ART) was introduced in the mid-1990s and has since led to a significant reduction in both morbidity and mortality associated with human immunodeficiency virus type 1 (HIV-1).^1^^,^^2^ While ART suppresses plasma viral loads (pVLs) to undetectable levels, it does not cure HIV-1. This is due to a pool of latent proviruses integrated into the host DNA, which can resume replication after the cessation of ART in the majority of people living with HIV-1 (PLWH) after just 16 days.^3^ Consequently, HIV-1 disease remains a chronic condition, and PLWH continue to face the risks of stigma, side effects and costs associated with ART.^4^^,^^5^

Efforts to cure HIV-1 have primarily focused on achieving ART-free immunological control either through the complete eradication of the HIV-1 reservoir or the induction of HIV-1-specific immunity.^6^ Several medical interventions are currently being investigated as potential means to achieve ART-free immunological control, including broadly neutralizing anti-HIV-1 antibodies (bNAbs), latency-reversing agents (LRAs), and toll-like receptor (TLR) agonists. Analytical treatment interruption (ATI) is considered the gold standard for assessing the effectiveness of such therapeutic interventions on ART-free immunological control.^7^ Before an ATI, participants are informed of the potential risks, which include the risk of HIV transmission, the development of drug resistance, and decreases in CD4^+^ T cell count.^8^ ATI participants are typically closely monitored with frequent pVL and CD4^+^ T cell count measurements until the criteria for resuming ART are met.^8^ These ART restart criteria vary significantly among trials; some studies define two consecutive measurements of pVL ≥200 copies/mL as viral rebound requiring ART resumption, while other trials have four to six weeks of sustained pVL ≥1000 copies/mL or two consecutive measurements of pVL ≥100,000 copies/mL as their threshold for ART resumption.^9^^,^^10^ In recent years, the criteria for restarting ART have become less stringent, permitting significantly higher pVLs for longer durations before resuming ART. The changes in ART restart criteria have raised concerns regarding the effect of peak pVL and duration of viremia on CD4^+^ T cell recovery after ART resumption.

However, the impact of the magnitude of viremia and the duration of virus exposure during ATI on CD4^+^ T cell recovery remains underexplored. This represents a significant gap in the field, as understanding this relationship is crucial for ensuring participant safety. Thus, we investigated the impact of peak pVL as well as duration of viremia on changes in CD4^+^ T cell count from pre-ATI to the time of ART resumption and viral re-suppression on ART across multiple trials conducted between 2012 and 2022 in which the sponsor and/or primary investigator were affiliated with Aarhus University Hospital.

## 2 Methods

The trials included in this analysis were conducted in accordance with the guidelines of the respective national competent authorities, and informed consent was obtained from all study participants (please refer to the individual studies for specific details).

### 2.1 Study design

We performed a pooled analysis of individual-level data collected from six interventional HIV-1 cure trials.^10^, ^11^, ^12^, ^13^, ^14^, ^9^ Participants were PLWH aged 18 years or older. In one of the six trials, participants were ART-naïve at enrolment; in the other five, they had received ART for at least one year.

### 2.2 Data extraction

The extracted data included age, sex, ethnicity, year of HIV-1 diagnosis, year of ART initiation, year of study enrolment, year of ATI, nadir CD4^+^ T cell count, along with longitudinal CD4^+^ T cell counts and pVL measurements from the pre-ATI time point, through the ATI to ART-induced viral re-suppression (defined as first plasma HIV-1 RNA <50 copies/mL).

### 2.3 Statistical analysis

Participant characteristics are presented as medians (ranges) or counts (percentages). Days with viremia were calculated based on longitudinal pVL measurements. If two consecutive time points showed pVLs exceeding the defined threshold (either >50 or >1000 copies/mL), the intervening days were considered viremic days. Conversely, if the pVL decreased below the threshold between two elevated measurements separated by time (e.g., suppression followed by a later rebound), only the confirmed viremia periods were regarded as viremic. This method was consistently applied to both the >50 and > 1000 copies/mL thresholds. pVL levels from the last visit of the ATI were used as the ART resumption measurement for individuals who maintained immunological control during the ATI. Time to loss of immunological control was defined as the number of days from stopping ART to either restarting ART or two consecutive measurements of plasma HIV-1 RNA ≥5000 copies/mL.

We used the Wilcoxon signed-rank test to evaluate differences in CD4^+^ T cell counts between pre-ATI and ART-induced viral re-suppression, as the data were paired but not normally distributed. The same analysis was conducted in subgroups stratified by pVL at ART resumption, with separate boxplots created for participants with pVL ≤10,000 copies/mL and >10,000 copies/mL. We used a linear regression model to analyze the relationship between pVL at ART resumption and time to loss of immunological control. The association between the change in CD4^+^ T cell count from pre-ATI to ART-induced viral re-suppression and the duration of viremia (0–14 days, 15–28, and >28 days) was assessed using the Wilcoxon rank-sum test. A *P* value of less than 0.05 was considered statistically significant. We used RStudio version 2023.06.2 + 561 software for statistical analysis.

## 3 Results

### 3.1 Study description

A total of 114 PLWH from six trials were included in the current analysis. Their baseline characteristics are summarized in Table 1. The median age ranged from 37 to 50 years across the trials. Most participants were male and Caucasian. All participants underwent an ATI at some point between November 2013 and June 2022. The trials investigated either single agents or combinations of agents, including bNAbs (3BNC117, 10–1074), LRAs (romidepsin (RMD), panobinostat (PNB)), a TLR9 agonist (MGN1703), or an HIV-1 peptide vaccine (Vacc-4x).

**Table 1:** Characteristics of the study population.

Characteristics	ROADMAP		CLEAR		REDUC B		TEACH B		eCLEAR		TITAN	
	(n = 17)		(n = 9)		(n = 16)		(n = 9)		(n = 20)		(n = 43)	
Age at inclusion	42	(33–62)	49	(28–51)	47	(32–62)	47	(37–57)	37	(22–68)	50	(24–62)
Female	3	(18)	0	(0)	2	(12)	0	(0)	2	(10)	6	(14)
Race and ethnicity												
Asian	0	(0)	0	(0)	1	(6)	0	(0)	2	(10)	0	(0)
Black or African	5	(29)	0	(0)	2	(13)	0	(0)	2	(10)	2	(5)
Caucasian	12	(71)	9	(100)	13	(81)	9	(100)	15	(75)	37	(86)
Other or more than one race	0	(0)	0	(0)	0	(0)	0	(0)	0	(0)	3	(7)
Unknown	0	(0)	0	(0)	0	(0)	0	(0)	1	(5)	1	(2)
Time from diagnosis to ART (years)	0	(0–10)	2	(0–18)	1	(0–9)	1	(0–25)	0	(0 - 0)	0	(0–21)
Time from diagnosis to baseline (years)	7	(2–31)	6	(2–27)	10	(3–27)	8	(2–31)	0	(0–1)	11	(2–38)
Time from ART to baseline (years)	7	(2–21)	3	(2–12)	6.5	(2–18)	6	(2–20)	0	(0–1)	9	(2–26)
Pre-ART Nadir CD4^+^ T cell count (cells/mm^3^)	NA		370	(179–710)	230	(60–710)	400	(29–690)	450	(242–950)	NA	

Data are median (range) or n (%).

ART, antiretroviral therapy; HLA, human leukocyte antigen; ATI, analytical treatment interruption; NA, not available.

The criteria for restarting ART during ATI varied among the trials (Supplemental Table 1). The ROADMAP trial had the strictest ART restart criteria, requiring two consecutive pVL measurements ≥200 copies per mL, whereas the criteria in the TITAN trial were less restrictive, with four weeks of sustained pVL ≥1000 copies/mL or two consecutive measurements of pVL >100,000 copies/mL.^9^^,^^10^

### 3.2 pVL trends during ATI

**Fig. 1:**
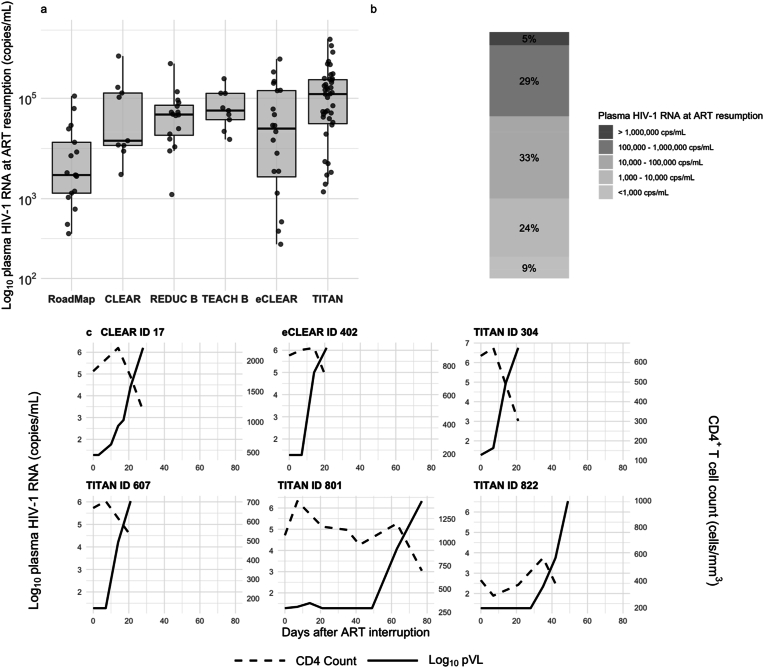
**Plasma viral load at ART resumption. a**, Distribution of plasma HIV-1 levels at ART resumption across six clinical trials: Roadmap (2017–2018), CLEAR (2012–2014), REDUC B (2014), TEACH B (2016–2017), eCLEAR (2020–2021), and TITAN (2021–2022). Trials are ordered from left to right based on the strictness of ART restart criteria. ART resumption criteria: Roadmap [>200 copies/mL], CLEAR [>1000 copies/mL], REDUC B [2x > 1000 copies/mL], TEACH B [2x > 5000 copies/mL], eCLEAR [2x > 5000 copies/mL], and TITAN [2x > 100,000 copies/mL or 4 weeks > 1000 copies/mL]. **b,** Percentage distribution of plasma HIV-1 RNA levels at ART resumption. **c,** CD4^+^ T cell and plasma HIV-1 RNA dynamics during ATI in individuals with plasma HIV-1 RNA >1,000,000 copies/mL. ART, antiretroviral therapy.

Fig. 1a illustrates trends in pVLs at ART resumption across the six included trials. Generally, more recent studies employed less restrictive ART resumption criteria, allowing for higher levels of viral rebound before reinitiating ART (Fig. 1b). This trend reflects a shift in trial design toward permitting longer periods of viral replication to evaluate the impact of interventions on immune-mediated immunological control (Fig. 1a + b). Five percent of the ATI participants (n = 6) had pVL >1,000,000 copies/mL at the time of ART resumption. Examining these participants individually, their virus rebounded extremely quickly to pVL >1,000,000 copies/mL at a median of 17 days (IQR: 14–25 days) following undetectable levels (Fig. 1c). None of these ATI participants experienced symptoms of acute retroviral syndrome. The six ATI participants achieved viral re-suppression at a median of 18 weeks (IQR: 16–19 weeks) following ART resumption.

### 3.3 Paired analysis of CD4^+^ T cell counts

**Fig. 2:**
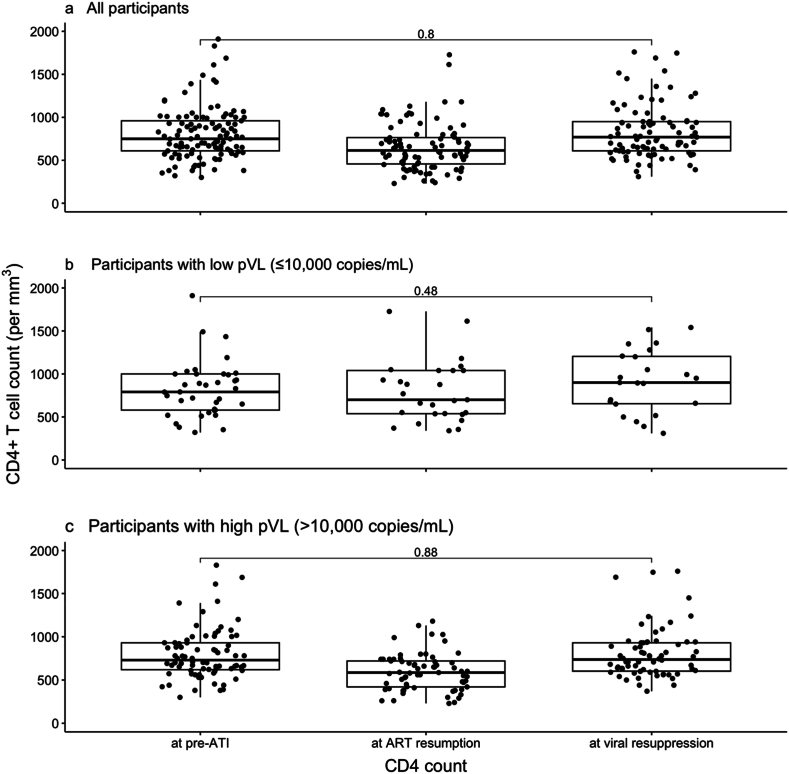
**CD4^+^ T cell counts at pre-ATI, ART resumption, and ART-induced viral re-suppression stratified by plasma HIV-1 RNA. a,** All participants. **b**, Participants with plasma HIV-1 RNA ≤10,000 copies/mL. c, Participants with plasma HIV-1 RNA >10,000 copies/mL. ART, antiretroviral therapy; ATI, analytical treatment interruption.

Next, we compared CD4^+^ T cell counts at pre-ATI, ART resumption, and ART-induced viral re-suppression. The first boxplot includes participant data (Fig. 2a), and no significant change in CD4^+^ T cell counts between pre-ATI and ART-induced viral re-suppression was observed (*P* = 0.80). Although six participants experienced a decline of more than 50 % in CD4^+^ T cell count during the ATI, none showed a sustained decrease of this magnitude when comparing pre-ATI levels to those at ART-induced viral resuppression. Additionally, seven participants experienced a drop in CD4^+^ T cell count to below 350 cells/μL during the ATI; however, all recovered to levels above 350 cells/μL after ART resumption. Participants were then stratified according to high (>10,000 copies/mL) versus low (≤10,000 copies/mL) pVLs at ART resumption (Fig. 2b + c). For both groups, the differences in CD4^+^ T cell counts at pre-ATI (*P* = 0.48) and ART-induced viral re-suppression (*P* = 0.88) were not statistically significant. These findings indicate that, irrespective of the magnitude of viral rebound during ATI, CD4^+^ T cell counts return to pre-ATI levels following viral re-suppression.

### 3.4 Association between pVL at ART resumption and time to loss of immunological control

**Fig. 3:**
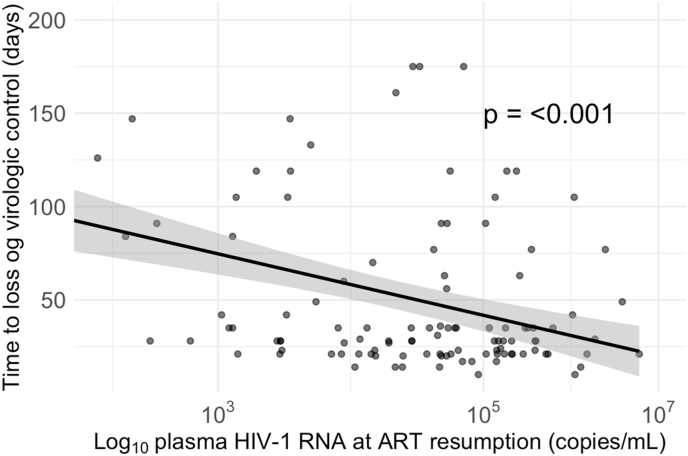
**Association between plasma HIV-1 RNA levels at ART resumption and time to loss of virologic control.** Linear regression model illustrating the association between Log_10_-transformed plasma HIV-1 RNA at ART resumption and days to loss of virologic control (defined as two consecutive plasma HIV-1 RNA measurements ≥5000 copies per mL or having not resumed ART during the ATI). ART, antiretroviral therapy; ATI, analytical treatment interruption.

We then examined the association between pVL at ART resumption and the time to loss of immunological control (Fig. 3). Our linear regression model indicated a negative association, suggesting that higher pVL at ART resumption is linked to a shorter duration until loss of immunological control (*P* < 0.001). The observed trend may suggest that individuals who restarted ART at higher levels of viremia also experienced a more rapid loss of immunological control compared to those with lower pVLs at resumption.

### 3.5 Impact of duration of viremia on CD4^+^ T cell count recovery

**Fig. 4:**
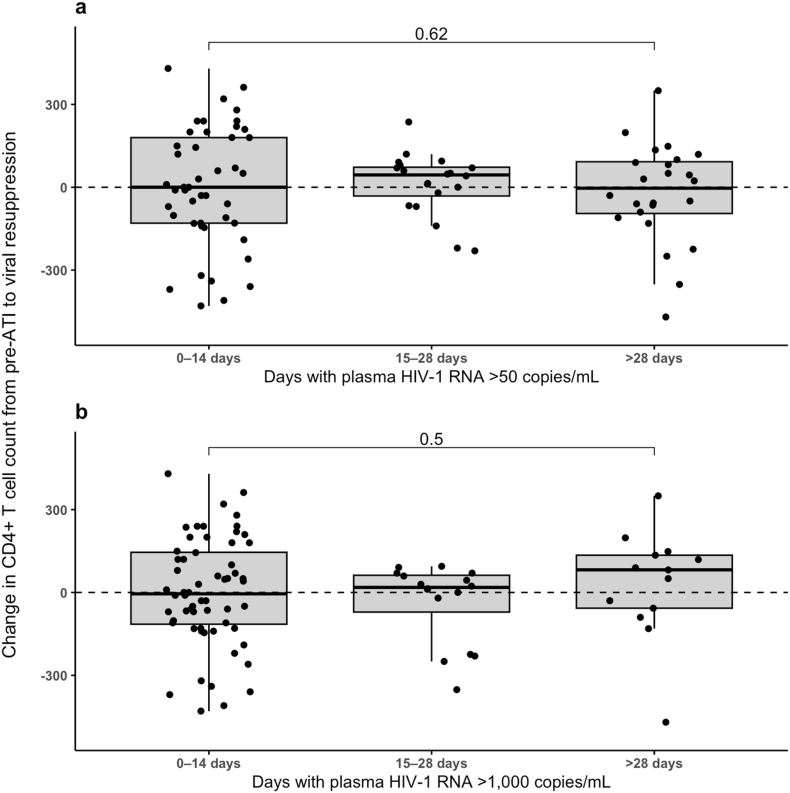
**Change in CD4^+^ T cell counts from pre-ATI to ART-induced viral re-suppression in relation to the duration of viremia. a,** Change in CD4^+^ T cell counts across three groups defined by the number of days with plasma HIV-1 RNA >50 copies/mL: 0–14 days, 15–28 days, and >28 days. **b,** Change in CD4^+^ T cell counts across three groups defined by the number of days with plasma HIV-1 RNA >1000 copies/mL: 0–14 days, 15–28 days, and >28 days.

Finally, we assessed the impact of the duration of viremia on changes in CD4^+^ T cell counts from pre-ATI to ART-induced viral re-suppression (Fig. 4). First, using a definition of viremia as pVL >50 copies/mL, we observed no significant impact of shorter (0–14 days) versus longer duration (>28 days) of viremia on CD4^+^ T cell recovery (*P* = 0.62). Similarly, when setting the threshold for viremia at >1000 copies/mL, we also found no significant difference in the change in CD4^+^ T cell counts across groups (*P* = 0.50), highlighting that at least for moderate periods of viremia, the duration of viremia does not impact CD4^+^ T cell recovery after ATI.

## 4 Discussion

Our findings demonstrate that neither peak viremia nor duration of viremia during ATI, impacted CD4^+^ T cell recovery after ART resumption in our pooled analysis. Thus, our results support the notion that controlled ATIs, when properly monitored, can be safely implemented in HIV cure clinical trials without significant risk of impaired CD4^+^ T cell recovery, even among individuals with peak pVL >1,000,000 copies/mL.

ATIs are a cornerstone of HIV cure research, enabling the evaluation of viral rebound dynamics and immune responses following ART cessation. Yet, despite their widespread use, the long-term immunological safety of ATIs remains a subject of debate. While some studies have suggested that transient viremia during ATI may not result in lasting harm to CD4^+^ T cell counts, others have raised concerns about potential delays or impairments in immune reconstitution.^15^^,^^16^ These conflicting findings highlight the need for further data to inform the safe design of ATI protocols. Our study addresses this gap by providing robust evidence from a large, well-characterized cohort of participants across multiple interventional HIV cure trials.

We further investigated the individual viral kinetics among the 5 % ATI participants with pVL exceeding 1,000,000 copies/mL at ART resumption, which aligns with the frequency observed in a data-based meta-analysis of 24 prospective ATI studies.^3^ In our pooled analysis, we noted that four of the six ATI participants were enrolled in the TITAN trial, where the ART restart criteria were less stringent. However, more stringent criteria would not have prevented pVL from exceeding 1,000,000 copies/mL, due to the rapid viral rebound kinetics. Reassuringly, these ATI participants re-suppressed after 18 weeks of ART, which is slightly longer than the 12 weeks of ART observed in a recent meta-analysis of interventional ATI studies.^17^ Previous studies suggesting that ATI-induced viremia may adversely affect immune recovery have primarily identified such effects in individuals with low CD4^+^ T cell counts prior to ART initiation or in those undergoing prolonged treatment interruptions.^18^^,^^19^ In these participants, the immune system may have had limited regenerative capacity, and extended periods of uncontrolled viremia likely contributed to further immune deterioration. These findings contrast with our results, which derive from a cohort with relatively high pre-ATI CD4^+^ T cell counts who underwent closely monitored, time-limited ATIs.

Other studies have indicated that short-term ATIs do not lead to significant long-term changes in CD4^+^ T cell counts. Clarridge et al. observed that while there was a transient expansion of the HIV-1 reservoir during ATI, CD4^+^ T cell levels returned to baseline after ART resumption.^15^ However, the study was based on a small cohort of only 10 individuals, which limits the generalizability of its findings. Similarly, another study reported comparable outcomes, noting an increased HIV-1 reservoir size during ATI, but no sustained impact on CD4^+^ T cell recovery following treatment resumption. Yet, as with the previous study, this research also had a limited sample size of only 22 individuals.^20^ Additionally, studies have demonstrated that transient viremia during ATI does not substantially affect the size or composition of the HIV-1 reservoir after ART is re-initiated.^21^^,^^22^ Early initiation of ART has been shown to restrict the establishment of viral reservoirs and reduce systemic inflammation by preventing monocyte infection; however, these cells are rapidly reinfected once ART is interrupted.^23^

Our study also had some potential limitations. The analyses were not adjusted for possible confounders such as therapeutic intervention or viral subtype, which could influence the estimates. Furthermore, the generalizability of our findings may be limited, as the included studies were conducted predominantly in white male populations, potentially reducing the external validity of our results. Finally, although our data suggest that ATI can be safely implemented even in individuals with pVL exceeding 1,000,000 copies/mL, this conclusion is based on a small subgroup of six participants and should therefore be interpreted with caution.

In conclusion, our findings provide essential evidence supporting the immunological safety of well-monitored, time-limited ATIs within the context of HIV-1 cure trials. This conclusion aligns with two meta-analyses on ATIs examining safety.^24^^,^^25^ Importantly, our findings emphasise the immunological safety of ATIs, whereas other studies focus on counselling, support, and psychosocial experiences of ATI participants and their partner(s).^26^^,^^27^ Our results contribute to an ongoing and critical discussion in the field by explicitly focusing on the relationship between ATI-induced viremia and CD4^+^ T cell recovery. They can help inform future ATI participants about what happens to their pVL and CD4 count once ART is paused. The relatively large cohort in our study enhances the robustness of our findings and supports the continued use of ATIs as a valuable tool in HIV-1 cure research.

## CRediT authorship contribution statement

**Vibeke Klastrup:** Writing – original draft, Visualization, Methodology, Funding acquisition, Formal analysis. **Jesper Damsgaard Gunst:** Writing – original draft, Visualization, Supervision, Methodology, Funding acquisition, Formal analysis, Data curation, Conceptualization. **Ole Schmeltz Søgaard:** Writing – original draft, Visualization, Supervision, Methodology, Funding acquisition, Formal analysis, Conceptualization.

## Statements and declarations

V.K., J.D.G., and O.S.S. wrote the protocol. V.K. and J.D.G. collected the data. V.K., J.D.G., and O.S.S. performed the statistical analysis, drafted the tables and figures, and composed the article, which all authors subsequently revised. None of the authors has conflicts of interest to declare.

## Declaration of generative AI and AI-assisted technologies in the writing process

During the preparation of this work the author(s) used OpenAI's ChatGPT for coding assistance. After using this tool/service, the author(s) reviewed and edited the content as needed and take(s) full responsibility for the content of the publication.

## Declaration of competing interest

The authors declare that they have no known competing financial interests or personal relationships that could have appeared to influence the work reported in this paper.

## Data Availability

Data will be made available on request.
