# Supplementary material for: The impact of magnitude and duration of plasma viremia during analytical treatment interruptions on CD4+ T cell recovery after ART resumption
Source: J Virus Erad. 2025 Jun 27;11(3):100604. doi: 10.1016/j.jve.2025.100604 (PMC12271902; doi:10.1016/j.jve.2025.100604)
Supplement: Multimedia component 1 [file mmc1.docx]

**Supplemental Table 1: Study characteristics**


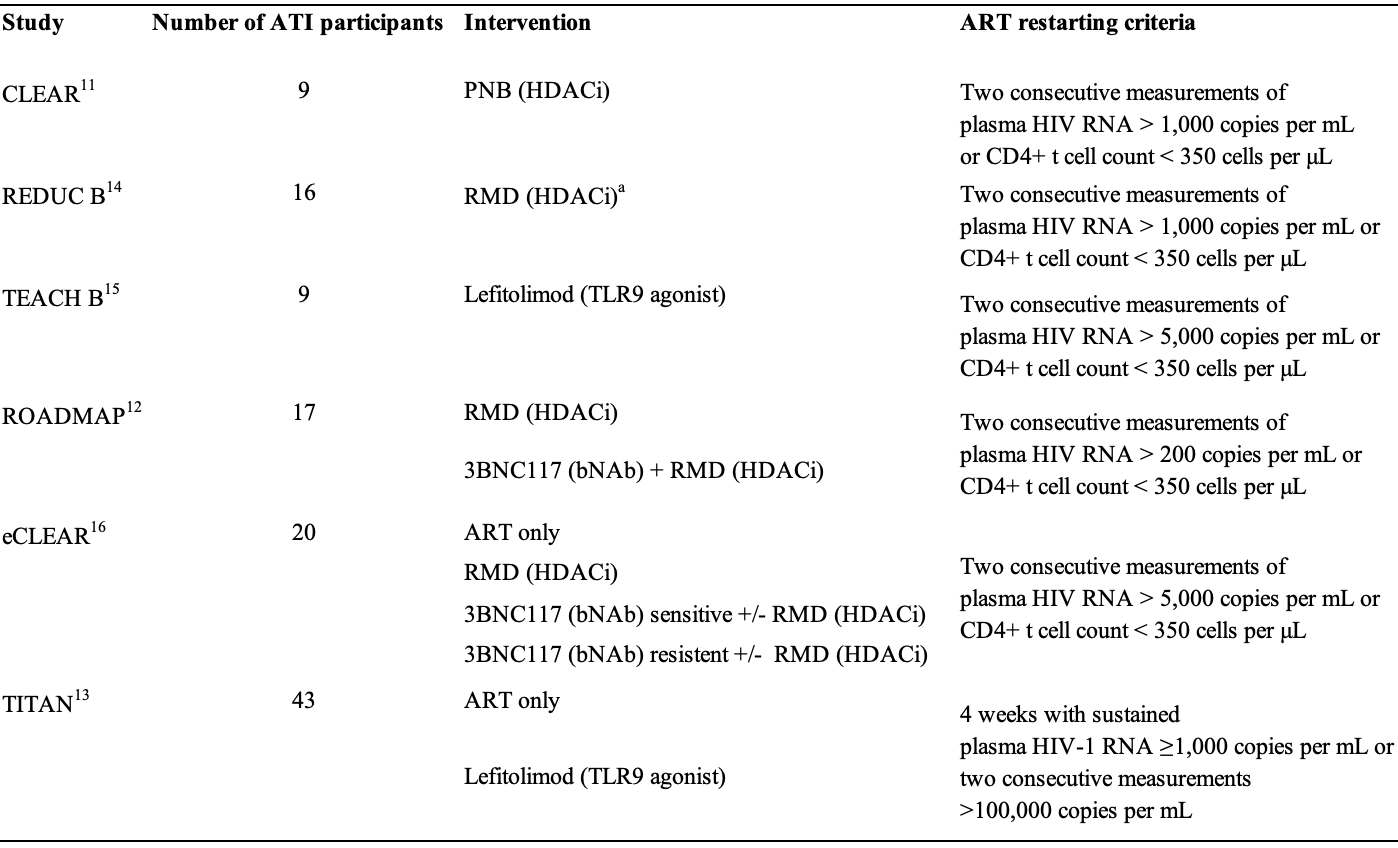


ART, antiretroviral therapy; ATI, analytical treatment interruption; HDACi, histone deacetylase inhibitor; PNB, panobinostat; RMD, romidepsin; TLR9 agonist, toll-like receptor 9 agonist; bNAb, broadly neutralising anti-HIV-1 antibodies. ^a^In the REDUC B study, participants received Vacc-4x in addition to RMD.
